# Irbesartan Ameliorates Lipid Deposition by Enhancing Autophagy via PKC/AMPK/ULK1 Axis in Free Fatty Acid Induced Hepatocytes

**DOI:** 10.3389/fphys.2019.00681

**Published:** 2019-05-29

**Authors:** Juan He, Jian Ding, Qiuhua Lai, Xinke Wang, Aimin Li, Side Liu

**Affiliations:** Guangdong Provincial Key Laboratory of Gastroenterology, Department of Gastroenterology, Nanfang Hospital, Southern Medical University, Guangzhou, China

**Keywords:** irbesartan, lipid deposition, autophagy, angiotensin II, PKC, AMPK, ULK1

## Abstract

Irbesartan has shown significant therapeutic effects in hypertensive patients with non-alcoholic fatty liver disease (NAFLD). To determine the underlying mechanisms of its action, we established an *in vitro* model of NAFLD by treating human and mouse hepatocytes with free fatty acids (FFAs) and angiotensin (Ang) II. Irbesartan significantly reversed AngII/FFA-induced lipid deposition and mitochondrial dysfunction by restoring ATP production and the mitochondrial membrane potential (MMP), and decreasing the levels of reactive oxygen species (ROS) and inflammatory markers. In addition, irbesartan also increased the autophagy flux, in terms of increased numbers of autolysosomes and autophagosomes, and the upregulation and mitochondrial localization of the autophagic proteins Atg5 and LC3BII/I. Activation of protein kinase C (PKC) and inhibition of the autophagic flux exacerbated mitochondrial dysfunction in the steatotic hepatocytes. Furthermore, AngII upregulated PKC which inhibited AMPK phosphorylation via direct interaction with the AngII receptor AT1-R. Irbesartan inhibited PKC and activated AMPK and its downstream effector ULK1, thereby inducing autophagy, decreasing lipid deposition, and restoring mitochondrial function. Taken together, irbesartan triggers autophagy via the PKC/AMPK/ULK1 axis to ameliorate the pathological changes in the steatotic hepatocytes.

## Introduction

Non-alcoholic fatty liver disease (NAFLD) affects approximately 25% of the global population, with steadily increasing rates worldwide (Younossi et al., 2016, 2017; Fan et al., 2017; Stefan, 2018). It includes a spectrum of hepatic pathologies, including non-alcoholic fatty liver, NASH and cirrhosis, and can even lead to hepatocellular carcinoma (HCC) (Kim et al., 2017; Eslam et al., 2018). NAFLD risk is increased by both genetic and environmental factors, and caused by lipid accumulation, oxidative stress, endoplasmic reticulum stress and insulin resistance at the molecular level. At present, the only effective therapy against NAFLD is adopting a healthier lifestyle (Serfaty, 2018). AngII, a key component of the RAS, has been implicated in the progression of NAFLD, with AngII type 1 receptor blockers (ARB) showing considerable therapeutic effects in patients with NAFLD/NASH (Nabeshima et al., 2009; Matthew Morris et al., 2013). We observed significant reduction in the levels of lipids and inflammatory markers in patients with both NAFLD and hypertension following ARB treatment. However, the underlying mechanisms of ARB action are still unclear.

A characteristic feature of NAFLD is steatosis caused by the accumulation of FFAs and TGs in the hepatocytes (Fabbrini et al., 2010). The hepatic lipid content is maintained through the balance between lipid uptake, synthesis, oxidation, and export. Excessive FFAs taken up by hepatocytes are converted into TGs and metabolized into DAG, which activates PKC and impairs the insulin signaling pathway (Perry et al., 2014). PKC inhibits the phosphorylation and activation of AMPK (Heathcote et al., 2016), which is related to the activation of ULK1 and autophagy (Kim et al., 2013). Lipid accumulation increases the generation of ROS as well as that of inflammatory cytokines. The “two hits theory” has long been considered the pathological basis of NAFLD and steatohepatitis (Day and James, 1998), proposing that steatosis is the first hit, and the development of steatohepatitis also requires a second hit, which is usually related with steatosis and can be a source of free radicals causing oxidative stress.

Although lipid accumulation is regarded as a consequence rather than a cause of insulin resistance, recent studies implicate steatosis as the primary step of NAFLD (Ioannou, 2016).

Autophagy is an evolutionarily conserved catabolic process essential for development and cell differentiation (Rautou et al., 2010), and involves recycling of damaged proteins and organelles for maintaining normal cellular homeostasis (Wong et al., 2017). Based on the pathway, it is classified into the predominant macroautophagy, microautophagy, and chaperone-mediated autophagy. Various stress stimuli, including starvation and ROS generation trigger macroautophagy, which is initiated by the formation of a primary double-membrane sequestering compartment known as phagophore. The latter expands into a spherical autophagosome enclosing various organelles like the mitochondria, endoplasmic reticulum and the Golgi complex, which then fuses with the lysosomal/vacuolar membrane forming the autolysosome, wherein the lysosomal permeases (Parzych and Klionsky, 2014) digest the cargo. Mitophagy refers to the autophagic degradation of mitochondria, and is essential for regulating the cellular ROS levels. One study showed that inhibition of autophagy in cultured hepatocytes and mouse liver increased TG storage in the form of lipid droplets (Singh et al., 2009). Lipophagy, i.e., degradation of lipids by autophagy is impaired in conditions involving lipid over-accumulation, such as NAFLD and NASH (Lavallard and Gual, 2014).

Several studies have shown a mechanistic role of RAS and AngII on hepatic fibrosis (Yoshiji et al., 2001; Yokohama et al., 2004), while those reporting the effect of ARB on NAFLD in murine models did not elucidate the underlying mechanisms (Sugimoto et al., 2006; Sturzeneker et al., 2011; Kato et al., 2012). AngII is a pro-inflammatory and pro-oxidant factor, and its receptor AT1-R is present on both normal and diseased liver parenchyma cells and associated with NAFLD (Yoneda et al., 2009; Liu et al., 2016). Irbesartan is a typical ARB, and has shown considerable therapeutic effects in both animal models and in hypertensive patients with NAFLD. The aim of this study was to dissect the precise mechanism of irbesartan action in NAFLD using an *in vitro* model consisting of FFA-treated hepatocytes.

## Materials and Methods

### Reagents and Antibodies

AngII (A9525), Sodium palmitate (P9767, PA), Sodium oleate (O7501, OA), NAC (A7250), Mito-Tempo (SML0737), and H_2_O_2_ (323381) were purchased from Sigma-Aldrich (United States), rapamycin (ab120224) from Abcam (United States), chelerythrine chloride (CHE, HY-12048) from MedChem Express (United States), and irbesartan (S1507) and 3-methyladenine (3-MA, S2767) from Selleck (United States). Antibodies against beclin1 (3459T), AMPKa (5831T), p-AMPKa (Thr172) (2535T), ULK1 (8054T), p-ULK1-Ser467 (4634T), p-ACC (Ser79) (11818T), ACC (3676T), PKC (2056T), ASC (67824T) and LC3B (3868) were purchased from Cell Signaling Technology (United States), NOX2 (sc-5827) and NOX4 (sc-21860) from Santa Cruz Biotechnology (United States), NLRP3 (MAB7578-SP) from R&D (United States), SQSTM1 (p62) (abs105873) from Absin (China), GAPDH (10494-1-AP) and β-actin (66009-1-Ig) from Proteintech (United States), and Atg5 (ab109490) and AT-1R (ab124505) from Abcam (United States).

### Cell Culture

The L02 cell line was obtained from the American Type Culture Collection (ATCC, Manassas, VA, United States), and Aml12 from the Chinese Academy of Sciences Cell Bank (Shanghai, China). Both cell lines were cultured in Dulbecco’s modified Eagle’s medium (DMEM) supplemented with 10% fetal bovine serum (FBS) at 37°C with 5% CO_2_. The FFA preparation consisted of a 1:2 mixture of palmitic acid (PA) and oleic acid (OA) with 1.1% bull serum albumin. The cells were cultured in the presence of PA, OA or the FFA mixture to establish an *in vitro* model of NAFLD, and further treated with AngII (10^-7^ M), irbesartan (10 nM), CHE (3 μM), NAC (0.01 M), mito-tempo (100 μM), rapamycin (50 μg/ml), or 3-MA (10 mM) as appropriate.

### Oil Red O Staining

Lipid deposition in the hepatocytes was assessed by oil red O staining. The dye solution was prepared in isopropanol and stored at 4°C. The cells were seeded onto glass slides and fixed in 4% paraformaldehyde for 30 min, followed by oil red O staining for 30 min and hematoxylin counterstaining for 10 min. The stained cells were imaged under a microscope using the Cellsens standard software (Olympus, Japan).

### Measurement of Intracellular Total Cholesterol (TC) and Triglyceride (TG) Levels

The cells were washed twice with phosphate buffer saline (PBS) and lysed with a lysis buffer. The intracellular TC and TG levels were measured using specific assay kits (E1015, E1013; Applygen Technologies Inc., China) according to the manufacturer’s instructions.

### Detection of Mitochondrial Membrane Potentials (MMPs)

Mitochondrial depolarization was evaluated using an MMP assay kit with JC-1 (C2006, Beyotime, China) according to the manufacturer’s instructions. The cells were cultured in 6-well plates, and incubated with 1 ml JC-1 staining solution (1×) at 37°C for 20 min. After rinsing twice with the staining buffer (1×), the presence of mitochondrial JC-1 monomers (green) and aggregates (red) were observed under a fluorescence microscope and laser confocal microscope (Olympus, Japan). Mitochondrial depolarization was indicated by an increase in the green/red fluorescence intensity ratio.

### Evaluation of ATP Levels

The ATP levels in the hepatocytes was measured using an ATP Assay Kit (S0026A, Beyotime, China) according to the manufacturer’s instructions. The cells were seeded in 6-well plates, treated with the requisite agents, lysed, and centrifuged at 12000 rpm for 5 min at 4°C. The supernatants were dispensed in opaque 96-well plates, and analyzed using the luminometer program of a microplate reader. The experiment was repeated thrice.

### Adenovirus Transfection and Immunofluorescent Cytochemistry

Cells were seeded into 24 well plates at 40–50% confluency, and transfected with tandem mRFP-GFP-LC3 adenovirus (Hanbio, China) at multiplicity of infection (MOI) of 50. After culturing for 6 h, the medium was replaced, and the cells were treated with AngII, FFA, or the agonists/inhibitors for 24 h. The treated cells were washed thrice with PBS and fixed with 4% paraformaldehyde for 10 min. The transfected cells were observed under a laser confocal microscope (Olympus, Japan), and the number of autolysosomes (red punctae) and autophagosomes (yellow due to merging of the red and green punctae) were counted. The experiment was repeated thrice.

### Measurement of Intracellular ROS and O_2_^-^ Levels

Intracellular ROS and O_2_^-^ generation were measured using 10 mM DCFH-DA (D6883, Sigma-Aldrich, United States) and DHE (S0063, beyotime biotechnology, China) probes, respectively. The cells were seeded into 96 well plates (5^∗^10^4^ cells per well), and incubated with 200 μl of either dye (diluted 1:1000 in serum-free medium) for 30 min at 37°C. After washing thrice with PBS (DCFH-DA) or serum-free medium (DHE), the fluorescence intensity was measured at the excitation and emission wavelengths of 488/525 nm for DCFH-DA and 300/610 nm for DHE using a microplate reader, and imaged under a fluorescence microscope (Olympus, Japan). Each sample was analyzed in quintuplets.

### Western Blotting

Hepatocytes were lysed on ice using a lysis buffer (Beyotime Biotechnology, China) supplemented with 1% PMSF and phosphatase inhibitors, and centrifuged at 12000 rpm for 30 min at 4°C. The supernatant protein levels were quantified with the BCA Protein Assay Kit (Thermo Fisher Scientific, United States), and equivalent amounts were resolved by SDS-PAGE. The protein bands were transferred onto PVDF membranes, and incubated overnight with primary antibodies (NOX2 and NOX4 diluted 1:200, rest 1:1000) at 4°C, followed by secondary antibody (1:1000) incubation for 2 h at room temperature. The immuno-positive bands were visualized by an X-ray film with an enhanced chemiluminescence substrate (Thermo Fisher Scientific, United States), and quantified by ImageJ.

### Co-immunoprecipitation (co-IP)

Total cellular proteins were extracted, and incubated with anti-AT-1R and anti-PKC antibodies (both diluted 1:10; IP group, line 1), anti-GAPDH antibody (1:10; negative control group, line 2), or 1X PBS (blank control group, line 3) for 10h at 4°C with constant shaking. Pre-washed protein-A-agarose (Roche, Switzerland) were added to the samples before overnight incubation at 4°C. The precipitates were collected by centrifuging at 3000 rpm and 4°C for 5 min, and washed by lysis buffer. The complexes were analyzed by Western blotting using suitable antibodies.

### Mitotracker Staining and Immunocytochemistry

The cells were seeded in 24-well plates, and after 24 h treatment with the different reagents, were incubated with pre-warmed 0.2 nM Mitotracker green (M7515, Invitrogen, United States) at 37°C for 30 min. After washing thrice with the buffer provided win the kit, the stained cells were fixed in 4% paraformaldehyde for 10 min and permeabilized with 0.2% triton X-100. The treated cells were incubated overnight with anti-Atg5 and anti-LCB3 (both diluted 1:200) at 4°C, followed by incubation with Alexa Fluor 555/488-conjugated anti-rabbit secondary antibody (1:200). After counterstaining with DAPI, the cells were observed under a laser confocal microscope (Olympus, Japan).

### Transmission Electron Microscopy (TEM)

Transmission electron microscope was used to detect changes in cellular membrane structure, lipid deposition and autophagy flux. Suitably treated cells were fixed in 2.5% glutaraldehyde (Sigma-Aldrich, United States), scraped, and centrifuged at 1000 rpm for 5 min. The cell pellets were fixed in 2.5% glutaraldehyde for 2 h at room temperature, processed further at 4°C as recommended, and observed by a TEM system (Wuhan Goodbio Technology, China).

### Statistical Analysis

All data are presented as mean ± SEM. Multiple groups were compared using the one-way ANOVA and two groups by the unpaired *t*-test. The graphs were prepared using Graphpad Prism version 7.0. *P*-values <0.05 were considered statistically significant.

## Results

### Irbesartan Suppresses Lipid Accumulation Induced by FFA and AngII in Hepatocytes

**FIGURE 1:**
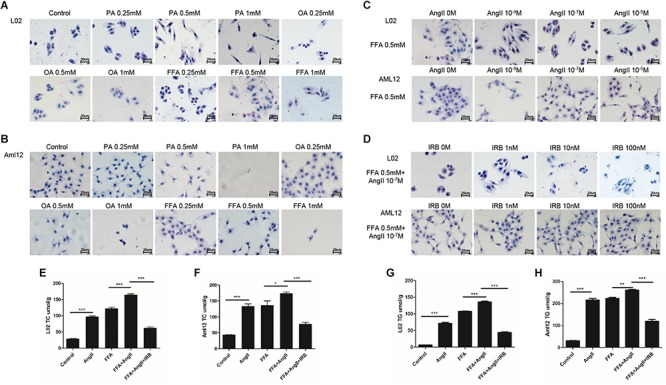
Irbesartan suppresses FFA and AngII-induced increase in lipid deposition and intracellular TG and TC levels in hepatocytes. Representative Oil red O-staining images showing dose-dependent effects of PA, OA, and FFA **(A,B)**, AngII **(C)**, and irbesartan **(D)** on lipid deposition in L02 and Aml12 cells. Magnification – 400×. **(E–H)** The effects of AngII and irbesartan on intracellular TC and TG levels. IRB, irbesartan; ^∗∗∗^*p* < 0.001; ^∗∗^*p* < 0.01; ^∗^*p* < 0.05. Values indicate means ± SEM, *n* = 3.

Lipid accumulation in the hepatocytes was simulated *in vitro* by treating the cell lines with varying amounts of PA, OA, and FFA, and lipid droplets were assessed by standard oil red O staining. Significant lipid deposition was seen in cells treated with 0.5 mM of the fatty acids without decrease in viability (Figure 1A,B), whereas higher concentrations induced cell death. PA was used for hepatocytes models in a previous study with 0.2 mM (Jiang et al., 2018), which is not enough for model establishment in our study. In addition, lower lipid accumulation was seen in PA-treated cells compared to the OA and FFA-treated cells, and the OA-treated L02 and Aml12 cells differed in the extent of lipid droplets. Therefore, 0.5 mM FFA was selected to induce steatosis in subsequent experiments. AngII also significantly increased lipid deposition in a dose-dependent manner (Figure 1C), with optimum effects seen at 10^-7^ M. Irbesartan significantly ameliorated FFA and AngII-induced lipid accumulation in a dose-dependent manner, and 10 nM was selected for subsequent experiments (Figure 1D). Furthermore, AngII significantly increased the intracellular levels of TC (Figure 1E,F) and TG (Figure 1G,H) in both the untreated and FFA-treated hepatocytes, which was downregulated by irbesartan (Figure 1E–H).

### Irbesartan Restores MMP and the Levels of ATP and Lipids in Hepatocytes by Inducing Autophagy

**FIGURE 2:**
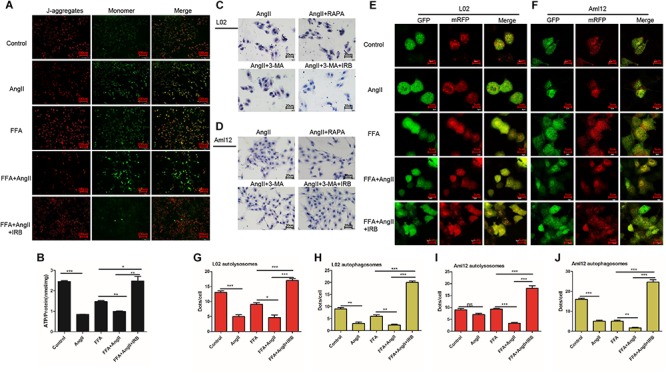
Irbesartan increases autophagy to reverse MMP depolarization, ATP decline and lipid accumulation in hepatocytes. **(A)** Representative JC-1-stained fluorescence images showing MMP changes in the AngII and irbesartan-treated cells. Magnification – 200×. **(B)** ATP levels in the AngII and irbesartan-treated cells. **(C,D)** Oil red O-staining images showing lipid deposition in AngII, irbesartan, rapamycin and 3-MA-treated L02 and Aml12 hepatocytes. Magnification – 400×. Values indicate means ± SEM, *n* = 3. **(E,F)** Representative fluorescence images showing autophagy flux in hepatocytes transfected with mRFP-GFP-LC3. Magnification – 1800×. **(G–J)** Number of autolysosome and autophagosome punctae in the differentially treated hepatocytes. RAPA, rapamycin; ^∗∗∗^*p* < 0.001; ^∗∗^*p* < 0.01; ^∗^*p* < 0.05. Values indicate means ± SEM, *n* = 3.

Mitochondrial membrane potential is an indicator of mitochondrial function, and its depolarization is measured in terms of increased fluorescence of JC-1 monomers (green) relative to its aggregates (red). While untreated hepatocytes showed little green fluorescence indicating normal MMP, the AngII-treated cells emitted strong green fluorescence indicating depolarization. In addition, AngII and FFA synergistically decreased the MMP compared to either reagent alone. Irbesartan restored AngII/FFA-depolarized membrane potential to near normal levels (Figure 2A). Interestingly, autophagy induction by rapamycin inhibited MMP depolarization, whereas inhibition of autophagy by 3-MA significantly increased the above compared to that in FFA and AngII co-treated cells (Supplementary Figure S1A), indicating that the autophagy flux reverses AngII-induced mitochondrial dysfunction.

AngII also significantly downregulated ATP levels in both untreated (AngII vs. control, *p* < 0.001) and FFA-treated hepatocytes (FFA+AngII vs. FFA, *p* = 0.0011) (Figure 2B), which was restored to normal by irbesartan (*p* = 0.003 compared to the FFA+AngII group; Figure 2B). Furthermore, autophagy induction re-established ATP production (Supplementary Figure S1B, *p* < 0.001 compared to FFA+AngII), while autophagy inhibition further decreased ATP levels (Supplementary Figure S1B, *p* < 0.001 compared to FFA+AngII). AngII-induced lipid accumulation was also inhibited by rapamycin but not significantly affected by 3-MA, whereas irbesartan blocked the impact of both 3-MA and AngII on hepatocyte steatosis (Figure 2C,D).

Based on these findings, we hypothesized that irbesartan relieves lipid accumulation, MMP depolarization and mitochondrial dysfunction by increasing the autophagy flux. To validate this hypothesis, we tracked the autophagy flux in the differentially treated hepatocytes using the mRFP-GFP-LC3 adenovirus (Figure 2E,F). The number of autolysosomes decreased significantly in the AngII-treated compared to the untreated L02 (*p* = 0.0006) and Aml12 (*p* = 0.0705) cells, and following AngII+FFA treatment compared to FFA stimulation alone (L02: *p* = 0.0147, Aml12: *p* = 0.0002). Irbesartan significantly increased the number of autolysosomes in both the FFA (L02: *p* = 0.0006, Aml12: *p* = 0.0020) and FFA+AngII treated (L02: *p* = 0.0003, Aml12: *p* = 0.0003) cells. AngII also reduced the autophagosomal count in the untreated (L02: *p* = 0.0002, Aml12: *p* = 0.0018 vs. untreated controls) and FFA-induced cells (L02: *p* = 0.0053, Aml12: *p* = 0.0075), which was increased by irbesartan (L02: compared with FFA, p < 0.001, with AngII, *p* < 0.001; Aml12: compared with FFA, *p* = 0.0001, with AngII, *p* < 0.001) (Figure 2G–J).

### Irbesartan-Induced Autophagy Relieves ROS and Inflammation in Hepatocytes

**FIGURE 3:**
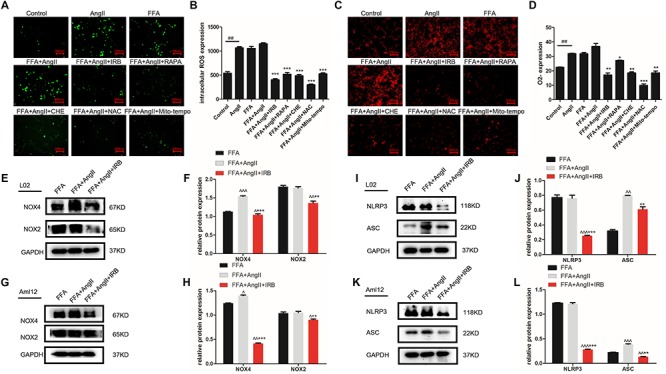
Autophagy flux induced by irbesartan relieves ROS and inflammation in hepatocytes. **(A,B)** Representative fluorescent images of DCFH-DA and DHE-stained hepatocytes treated with AngII, irbesartan, rapamycin, CHE, NAC, and mito-tempo. Magnification – 200×. **(C,D)** Fluorescence intensities of the ROS probes in the differentially treated cells. Immunoblots showing expression levels of NOX4 and NOX2 in L02 **(E,F)** and Aml12 **(G,H)** cells treated with irbesartan. Immunoblots showing expression levels of NLRP3 inflammasome, NLRP3 and ASC in L02 **(I,J)** and Aml12 **(K,L)** cells treated with irbesartan. ^##^*p* < 0.01, vs. Control. ^∗∗∗^*p* < 0.001, ^∗∗^*p* < 0.01, ^∗^*p* < 0.05, vs. FFA+AngII; ^∧∧∧^*p* < 0.001, ^∧∧^*p* < 0.01, ^∧^*p* < 0.05, vs. FFA.

Lipid deposition in hepatocytes results in mitochondrial dysfunction, increased ROS generation and activation of the inflammatory response. AngII significantly increased the ROS and O_2_^-^ levels in hepatocytes, as indicated by the decreased fluorescence intensity of the DCFH-DA (Figure 3A,B; *p* = 0.0002 vs. control) and DHE probes (Figure 3C,D; *p* < 0.0001 vs. control), while irbesartan significantly weakened the fluorescence intensity (DCFH-DA: *p* < 0.0001; DHE: *p* = 0.0057). In addition, irbesartan also downregulated the oxidative enzymes NOX4 and NOX2 in the steatotic hepatocytes (Figure 3E–H). PKC inhibition by CHE (DCFH-DA: *p* < 0.0001; DHE: *p* = 0.0062) and autophagy induction (DCFH-DA: *p* = 0.0001; DHE: *p* = 0.0332) significantly decreased the ROS levels in the AngII+FFA treated hepatocytes, indicating that irbesartan likely exerts its antioxidant effects via the PKC/autophagy axis. The expression of the inflammasome components NLRP3 and ASC were also alleviated by irbesartan in both L02 (Figure 3I,J) and Aml12 (Figure 3K,L), underscoring the involvement of inflammation in irbesartan action.

### Irbesartan Induces Autophagy by Downregulating PKC and Activating the AMPK/ULK1 Axis

**FIGURE 4:**
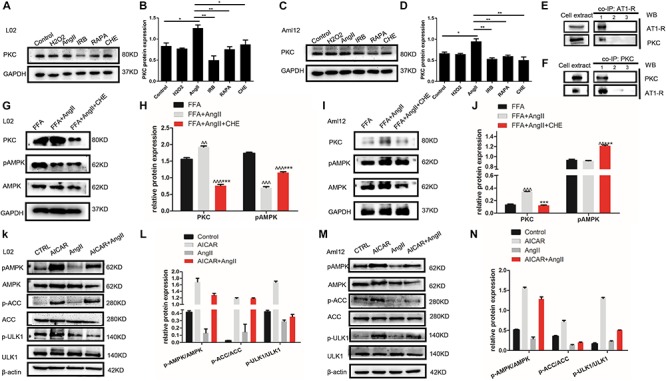
AngII upregulates PKC and inhibits the phosphorylation of AMPK and ULK1. **(A–D)** Expression levels of PKC in AngII, irbesartan, CHE, and rapamycin-treated hepatocytes. ^∗∗^*p* < 0.01, ^∗^*p* < 0.05. **(E,F)** Co-IP of AT1-R and PKC in the presence of anti-PKC **(E)** and anti-AT1-R **(F)** antibodies. **(G–J)** Expression levels of pAMPK in CHE-treated L02 and Aml12 hepatocytes. ^∧∧∧^*p* < 0.001, ^∧∧^*p* < 0.01, vs. FFA. ^∗∗∗^*p* < 0.001 vs. FFA+AngII. **(K–N)** Expression levels of AMPK, ACC, and ULK1 in AICAR and AngII-treated L02 and Aml12 hepatocytes.

To further dissect the role of autophagy in irbesartan-mediated effects, we first analyzed the expression levels of PKC. AngII significantly upregulated PKC in the hepatocytes (L02: *p* = 0.0127, Aml12: *p* = 0.0152), which were decreased by CHE (L02: *p* = 0.0385, Aml12: *p* = 0.0099), irbesartan (L02: *p* = 0.0034, Aml12: *p* = 0.0035), and rapamycin (L02: *p* = 0.0058, Aml12: *p* = 0.0066) (Figure 4A–D). Furthermore, co-IP experiments showed a direct interaction between AT1-R, the typical receptor of AngII, and PKC (Figure 4E,F). AngII increased PKC levels and downregulated p-AMPK (Thr-172) in the hepatocytes (Figure 4G–J), whereas inhibition of PKC increased AMPK phosphorylation, thus reversing the effects of AngII (Figure 4G,I). Taken together, irbesartan downregulated PKC and activated AMPK in the hepatocytes by blocking the AngII/AT1-R axis.

**FIGURE 5:**
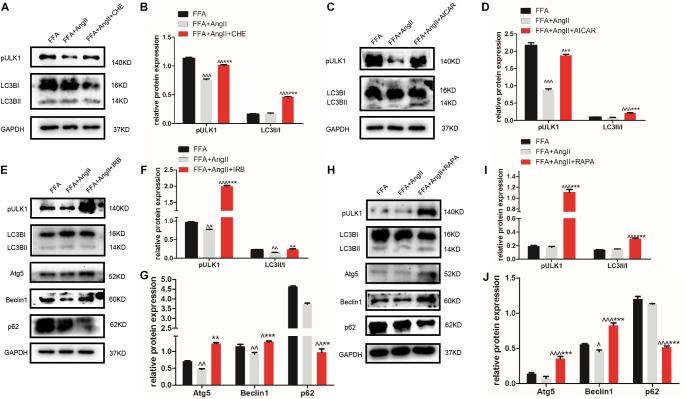
Irbesartan activates autophagy by downregulating PKC and activating the AMPK/ULK1 axis. **(A–D)** Expression levels of pULK1-s467 and LC3BII/I in CHE and AICAR-treated hepatocytes. **(E–J)** Expression levels of pULK1-s467, LC3B, Atg5, beclin1, and p62 in hepatocytes treated with irbesartan and rapamycin. ^∧∧∧^*p* < 0.001, ^∧∧^*p* < 0.01, ^∧^*p* < 0.05, vs. FFA. ^∗∗∗^*p* < 0.001, ^∗∗^*p* < 0.01, vs. FFA+AngII.

Furthermore, AngII also inhibited the action of the AMPK agonist AICAR on the phosphorylation of AMPK and ACC, and AMPK’s downstream target ULK1 (Figure 4K–N), the activation of which is the initiating step of autophagy. Inhibition of PKC (Figure 5A,B) as well as AICAR-mediated activation of AMPK (Figure 5C,D) significantly increased both ULK1 phosphorylation and LC3BII/I ratio, indicating autophagy induction. Both irbesartan (Figure 5E–G) and rapamycin (Figure 5H–J) reversed the effects of AngII on PKC and AMPK, which upregulated the pro-autophagy proteins. Taken together, AngII downregulated autophagy by inducing PKC and deactivating AMPK, and irbesartan-mediated blockade of the AngII/AT1-R/PKC axis participated in activating AMPK and ULK1, thereby playing a role in increasing the autophagy flux.

### Irbesartan-Induced Autophagy Alleviates Steatosis and Mitochondrial Dysfunction in Hepatocytes

**FIGURE 6:**
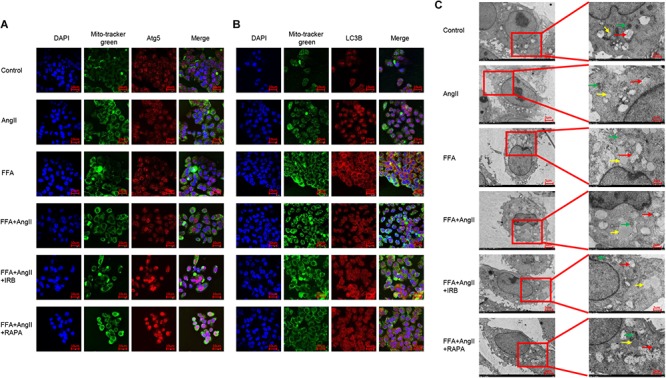
Irbesartan activates mitophagy to relieve steatosis in hepatocytes. **(A,B)** Representative immunofluorescence images showing co-localization of mito-tracker green with Atg5 and LC3B in differentially treated hepatocytes. **(C)** TEM images showing the ultrastructure of lipids (yellow arrows), autophagosome (red arrows), and mitochondria (green arrows). Magnification: left lane – 2000×; right lane – 5000×.

To track the mitochondrial localization of the autophagy-related proteins Atg5 and LC3B in the differentially treated hepatocytes, we co-stained the cells with Mitotracker and the specific antibodies. AngII and FFA inhibited the mitochondrial localization of Atg5 (Figure 6A) as well as LC3B (Figure 6B). Irbesartan and rapamycin not only increased the levels of Atg5 and LC3B, but also their mitochondrial localization (Figure 6A,B), indicating an autophagic flux and mitochondrial phagocytosis. Ultrastructural examination by TEM showed that the lipids droplets and dysfunctional mitochondria were engulfed by the autophagosomes and autolysosomes. The number of autophagosomes were significantly decreased and that of lipidosomes increased in the AngII-treated cells, and these changes were reversed by irbesartan. The latter also decreased the excessive liposome deposition induced by FFA and AngII co-treatment (Figure 6C).

## Discussion

Hepatocyte lipid deposition is the initiating step and hallmark in NAFLD pathogenesis. Hepatic lipotoxicity refers to the cellular injury and death caused by the exposure to, or accumulation of FFAs and triglyceride within hepatic cells (Neuschwander-Tetri, 2010). Lipotoxicity leads to the development of hepatic inflammation and fibrosis in patients with NAFLD (Ioannou, 2016), which can further progress to NASH, hepatic cirrhosis and even HCC. And we should be aware of the close link between lipotoxicity and NAFLD, type 2 diabetes mellitus and cardiovascular disease (Cusi, 2012). Cholesterol accumulation indicates the dysregulation of liver cholesterol homeostasis in hepatocytes, and excessive cholesterol can lead to the development of NASH in animal models (Ioannou, 2016). Lipid accumulation is closely associated with the manifestations of the metabolic syndrome, such as hypertension, hyperglycemia, hyperlipidemia, and hyper-insulin resistance. NAFLD is a hepatic manifestation and a risk factor of metabolic syndrome with type 2 diabetes mellitus, dyslipidemia, and hypertension (Kawano and Cohen, 2013).

The RAS is a typical therapeutic target of hypertension, and its classical axis is mediated by AT1-R and related with inflammation and ROS. In addition, studies also show a crucial role of the RAS/AngII/AT1 pathway in the development of NAFLD (Yang et al., 2005). Irbesartan, an important subset of AngII receptor blockers, is commonly used as antihypertensive drugs. The therapeutic effects of irbesartan on hypertension and hyperlipidemia indicate a potential ameliorative role in hepatic steatosis as well. Thus irbesartan can be a progress for better clinical combination therapy if the underlying mechanism is proved.

Indeed, irbesartan was reported to relieve steatosis in db/db mice by activating the AMPK/Akt/mTOR pathway and inducing autophagy (Zhong et al., 2017), and the results was also a proof that irbesartan regulated autophagy in ameliorating lipid accumulation. ACC, the sustrate of AMPK, plays a role in lipid regulation, indicates AMPK a potential target in lipid accumulation as well. Also, there was reports that AngII might play a role in inducing autophagy in other cells such as podocytes (Yadav et al., 2010) while cellular homeostasis differed in different stages of cells and diseases. The aim of this study therefore was to explore the role of irbesartan in regulating the AngII/AT1-R axis in an *in vitro* model of hepatic steatosis (Jiang et al., 2018). AngII aggravated lipid accumulation in the FFA-treated hepatocytes resulting in oxidative stress and inflammation, indicating a pivotal role of AngII and irbesartan in NAFLD pathogenesis.

Protein kinase C is activated by DAG and mediates ROS production in blood cells (George et al., 2013). Lipid infusion further activates PKC (Greene et al., 2010), whereas genetic deletion of PKC is known to block NADPH oxidase activity (Greene et al., 2014). Inhibition of PKC has also been associated with the activation of autophagy (Wong et al., 2017), as well as increased AMPK activity via de-phosphorylation of the Ser487 residue (Heathcote et al., 2016). We found that PKC also targeted AMPK at Thr172, and blocked its activation. In addition, PKC was significantly upregulated by AngII, and its direct interaction with AT1-R increased the levels of ROS and inflammatory factors. Irbesartan ameliorated the effect of AngII by downregulating PKC, resulting in reduced steatosis, oxidative stress and inflammation. Taken together, irbesartan exerted its effects by inhibiting PKC and thus activating the downstream pathway driving autophagy flux.

Autophagy protects cells against various stresses by maintaining intracellular homeostasis and ensuring adequate energy levels (Mizushima et al., 2008). Autophagy promotes cell survival via removing damaged organelles or cellular proteins by lysosomal degradative pathway, which is suggested to mediate the breakdown of intracellular lipids in hepatocytes (Czaja, 2016). Thus autophagy may participate in regulation of hepatic steatosis and NAFLD. Inhibition of autophagy in hepatocytes and mouse liver increased TG levels and lipid droplets (Singh et al., 2009; Ding et al., 2010), indicating its critical role in regulating lipid metabolism.

**FIGURE 7:**
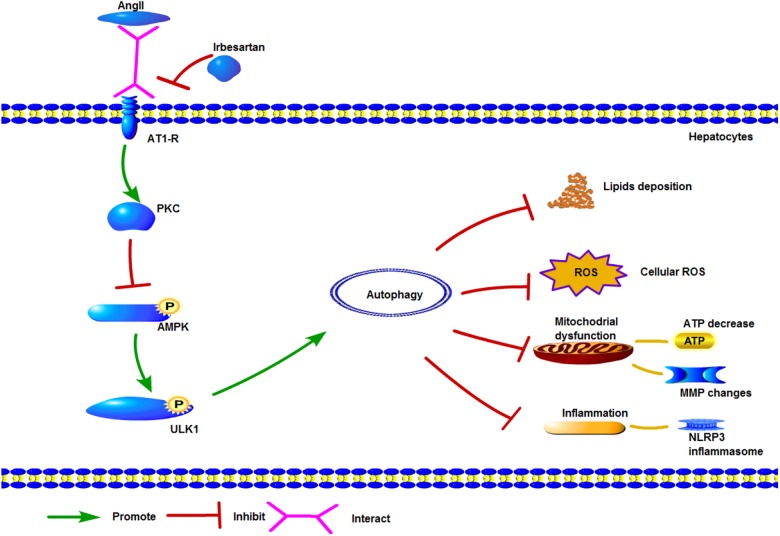
Graphical model of irbesartan-mediated induction of autophagy via the PKC/AMPK/ULK1 axis.

ULK1, the initiator of autophagy, activates beclin1 during autophagic vacuole formation (Rabinowitz and White, 2010; Rautou et al., 2010). It is activated by AMPK (Wong et al., 2013), which can sense the cellular energy status and the AMP/ATP ratio, and is often dysregulated in metabolic diseases (Day et al., 2017). AMPK can directly phosphorylate ULK1, as well as indirectly activate ULK1 by inhibiting mammalian target of rapamycin (mTOR) (Lin and Hurley, 2016). ULK1 in turn phosphorylates beclin1 at ser30 thereby activating the protein (Park et al., 2018), and both ULK1 and AMPK regulate the VPS 34 complex (Kim et al., 2013; Russell et al., 2013). AngII inhibited the phosphorylation of AMPK, ACC, and ULK1, and blocked the initiation of autophagy, while AMPK activation by its specific agonist AICAR circumvented this AngII-mediated inhibition. Furthermore, irbesartan activated ULK1 via phosphorylation at ser467, and promoted the autophagy flux, as indicated by increased expression levels of LC3BII/I, beclin1 and Atg5. Autophagy can restore the homeostasis in steatotic hepatocytes by engulfing the lipid droplets and dysfunctional mitochondria. Irbesartan initiated the autophagy flux and relieved steatosis by modulating the PKC/AMPK/ULK1 axis (Figure 7), thus both autophagy and lipophagy are dynamic processes in hepatocytes. Lipid homeostasis is repaired by irbesartan induced autophagy as lipid droplets are degraded by autophagosomes and autolysosomes. Irbesartan has long been an effective antihypertensive drug and based on our findings, can also be used as an anti-hyperlipidemic agent in anti-NAFLD therapy, after further validation in animal models and clinical studies.

## Author Contributions

JH designed and performed the experiments, analyzed the results, and drafted the manuscript. JD performed the experiments and prepared the figures. QL and XW helped with data analysis. AL guided JH and JD with the experiments. SL guided manuscript writing.

## Conflict of Interest Statement

The authors declare that the research was conducted in the absence of any commercial or financial relationships that could be construed as a potential conflict of interest.

## Supplementary Material

FIGURE S1 Autophagy flux induced by irbesartan relieves mitochondrial damage in hepatocytes. **(A)** Results of JC-1 probe was applied to confirm MMP with application of AngII, irbesartan, rapamycin and 3-MA. Magnification: ×200. **(B)** Results of ATP level with AngII, irbesartan, rapamycin and 3-MA. ^∗∗∗^*p* < 0.001, vs FFA+AngII group.

## Abbreviations

3-MA: 3-methyladenine
AMPK: adenosine monophosphate activated protein kinase
AngII: angiotensin II
ARB: angiotensin II type 1 receptor blocker
CHE: chelerythrine chloride
co-IP: co-immunoprecipitation
DAG: diacylglycerol
DCFH-DA, 2: 7-dichlorodihydrofluorescein diacetate
DHE: dihydroethidium
FFA: free fatty acid
MMP: mitochondrial membrane potential
NAC: *N*-Acetyl-L-cysteine
NAFLD: non-alcoholic fatty liver disease
NASH: non-alcoholic steatohepatitis
OA: sodium oleate
PA: sodium palmitate
PBS: phosphate buffer
PKC: protein kinase C
RAS: renin–angiotensin system
ROS: reactive oxygen species
TC: total cholesterol
TEM: transmission electron microscope
TG: triglycerides
ULK1: unc-51 like autophagy activating kinase 1
