# Supplementary figures and images for: Irbesartan Ameliorates Lipid Deposition by Enhancing Autophagy via PKC/AMPK/ULK1 Axis in Free Fatty Acid Induced Hepatocytes
Source: Front Physiol. 2019 May 29;10:681. doi: 10.3389/fphys.2019.00681 (PMC6548903; doi:10.3389/fphys.2019.00681)

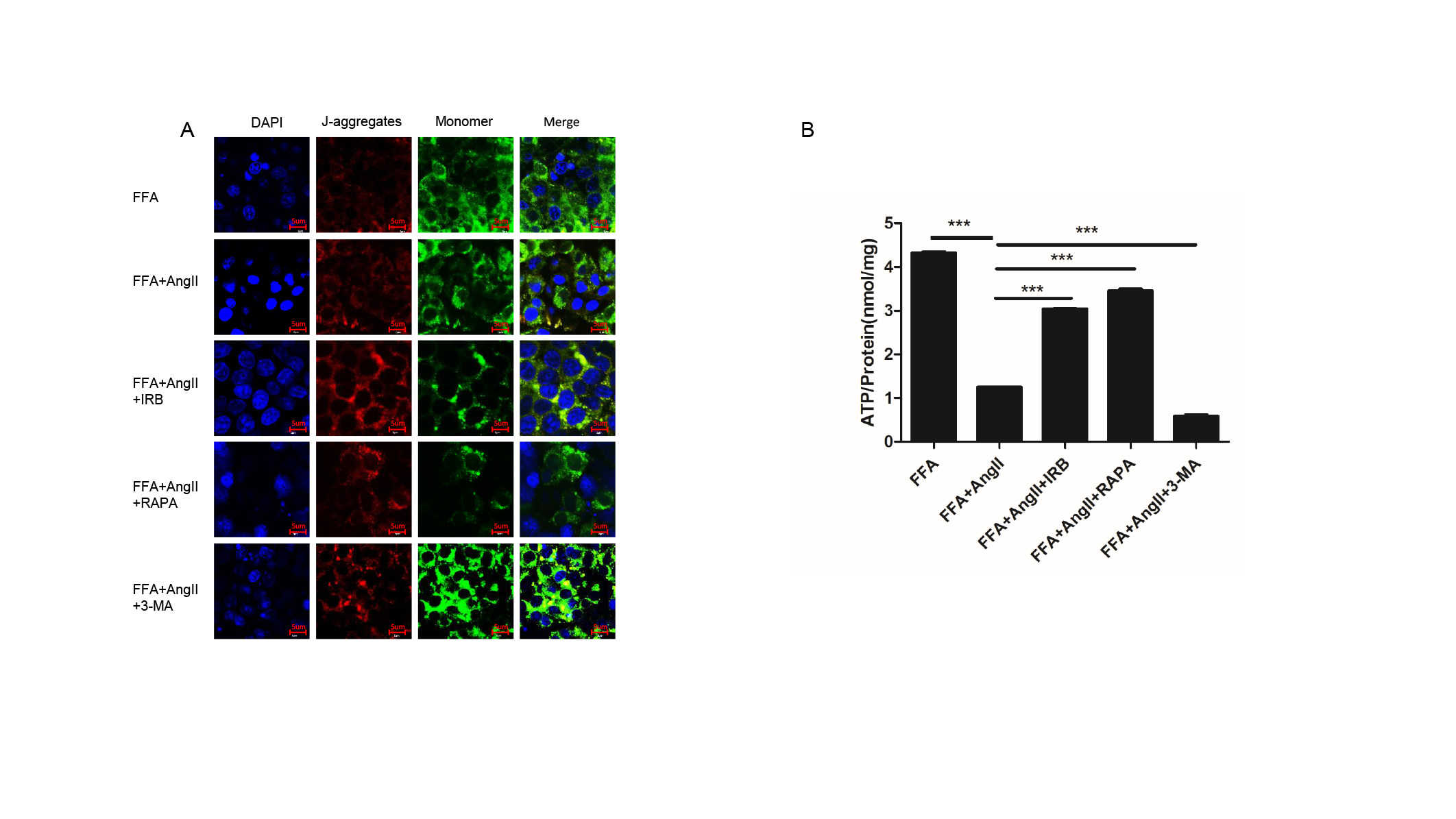

Supplement: FIGURE S1 — Autophagy flux induced by irbesartan relieves mitochondrial damage in hepatocytes. (A) Results of JC-1 probe was applied to confirm MMP with application of AngII, irbesartan, rapamycin and 3-MA. Magnification: ×200. (B) Results of ATP level with AngII, irbesartan, rapamycin and 3-MA. ∗∗∗p < 0.001, vs FFA+AngII group. [file Image_1.TIF]
